# Neuroprotective and Cytotoxic Phthalides from *Angelicae Sinensis* Radix

**DOI:** 10.3390/molecules21050549

**Published:** 2016-04-26

**Authors:** Wenxia Gong, Yuzhi Zhou, Xiao Li, Xiaoxia Gao, Junsheng Tian, Xuemei Qin, Guanhua Du

**Affiliations:** 1 Modern Research Center for Traditional Chinese Medicine, Shanxi University, No.92, Wucheng Road, Taiyuan 030006, China; 2 Institute of Materia Medica, Chinese Academy of Medical Sciences & Peking Union Medical College, Beijing 100050, China

**Keywords:** phthalides, *Angelicae Sinensis* Radix, cytotoxicity activity, flow cytometry, neuroprotective effect

## Abstract

Seven phthalides, including a new dimeric one named tokinolide C (**7**), were isolated from *Angelicae Sinensis* Radix and characterized. The structures of these compounds were elucidated on the basis of comprehensive analysis of spectroscopic data and comparison with literature data. All of the compounds were evaluated for their cytotoxic activities against the A549, HCT-8, and HepG2 cancer cell lines. Riligustilide (**4**) showed cytotoxicity against three cancer cell lines, with IC_50_ values of 13.82, 6.79, and 7.92 μM, respectively. Tokinolide A (**6**) and tokinolide C (**6**) exerted low cytotoxicity in these cancer cell lines, while the remaining compounds were inactive. Flow cytometry analysis was employed to evaluate the possible mechanism of cytotoxic action of riligustilide (**4**). We observed that compound **4** was able to arrest the cell cycle in the G1, S phases and induce apoptosis in a time-dependent manner in HCT-8 cell lines. In addition, these compounds were evaluated for neuroprotective effect against SH-SY5Y cells injured by glutamate. The result showed that ligustilide (**1**), *Z*-butylidenephthalide (**3**) and tokinolide A (**6**) exhibited significant neuroprotective effects.

## 1. Introduction

The roots of *A. sinensis* (Oliv.) Diels, also known in Chinese as Dang Gui, a well-known herb belonging to the Umbelliferae family, has been used for more than 2000 years in China. Their ability to produce structurally diverse and biologically significant monomeric and dimeric phthalide metabolites is well documented [1,2]. Previous pharmacological activity studies revealed that these compounds display anti-proliferative [3,4], anti-inflammatory [5,6,7], anti-thrombus [8], neuroprotective [9,10,11], anti-tumor [12,13], and anti-spasmodic effects. In recent years, the bioactivities of these compounds have attracted a great interest and extensive research has been carried out to characterize the molecular mechanism(s) behind their activity. However, the studies on the bioactivities of these compounds have been mainly focused on just the monomeric phthalides. In ongoing research efforts to discover novel and bioactive phthalides from *Angelicae Sinensis* Radix, seven phthalides including three monomeric phthalides and together with four dimeric phthalides were obtained herein. These phthalides were evaluated for cytotoxicity against the A549 (human lung carcinoma), HCT-8 (human colon carcinoma), and HepG2 (human liver carcinoma) cancer cell lines. The possible mechanism of cytotoxicity action of riligustilide (**4**) was also evaluated by flow cytometry analysis. Numerous studies have reported that glutamate-induced neuronal death is associated with the development of several neurodegenerative diseases including Alzheimer’s disease, Parkinson′s disease, and amyotrophic lateral sclerosis [14,15] and it had been widely used in the drug research. In this study, we used an *in vitro* model of Glu-induced excitotoxicity in SH-SY5Y cells to investigate the neuroprotective functions of phthalides. The results revealed that ligustilide (**1**), *Z*-butylidenephthalide (**3**) and tokinolide A (**6**) could significantly antagonize the neurotoxicity of glutamate, suggesting that these compounds could serve as candidates for potential use in the treatment of neurodegenerative diseases.

## 2. Results and Discussion

### 2.1. Isolation, Identification, and Structure Elucidation

**Figure 1:**
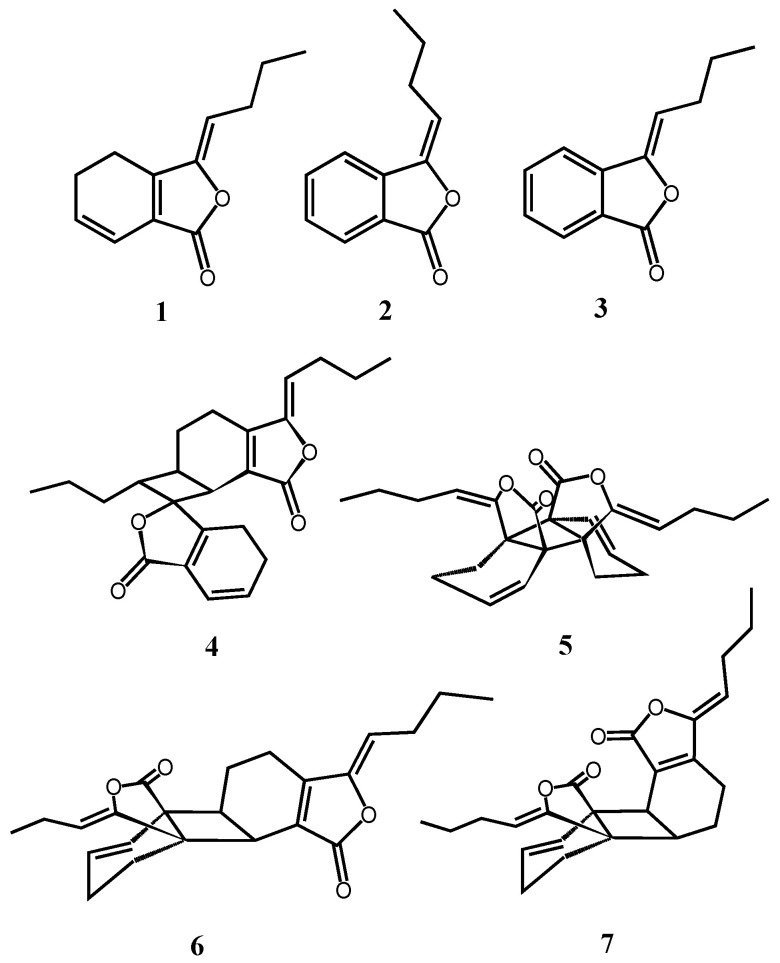
Chemical structures of the isolated compounds **1**–**7**.

The supercritical fluid CO_2_ extract was subjected to successive column chromatography (CC) on silica gel (SiO_2_) and Sephadex LH-20 gel columns, and to reversed-phase HPLC, which led to the isolation of seven monomeric and dimeric phthalides, including a new compound, named tokinolide C (Figure 1). Among these compounds, five known compounds were identified as ligustilide (**1**) [16], *E*-butylidenephthalide (**2**) [17], *Z*-butylidenephthalide (**3**) [17], riligustilide (**4**) [18], *cis*-Z,Z′-3a. 7a′,7a. 3a′-dihydroxyligustilide (**5**) [19] by ^1^H-NMR, and ^13^C-NMR spectroscopic data comparison with the corresponding literature data. The structures of the new compound (**7**) and tokinolide A (**6**) were elucidated on the basis of spectroscopic data, and their proposed structures were supported by analysis of the corresponding HMQC, HMBC spectra. The relative configuration of **7** was assigned by a NOESY experiment. Models of the molecule of **7** were optimized by MM2 energy-minimized three-dimensional molecular mechanics using Chem 3D Ultra 9.0.

**Figure 2:**
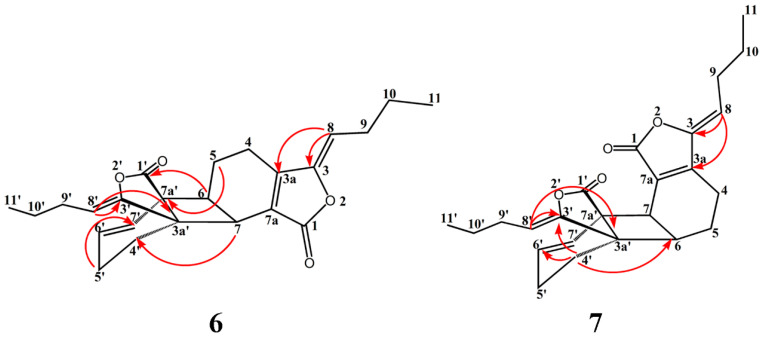
Selected HMBC correlations of compound **6** and **7**.

Compound **6** was isolated as a pale yellow oil. The structure were confirmed by ^1^H-NMR and ^13^C-NMR spectroscopic data. The HSQC, HMBC experiments allowed for the full assignment of the NMR signals of **6**. The ^1^H-NMR data (Table 1) exhibited signals corresponding to the presence of four olefinic protons, two methines and two methyl groups. Analysis of the ^13^C-NMR spectra showed that compound **6** possessed twenty-four carbon resonances including two carbonyls, eight olefinic carbons, two methyls. Bidirectional HMBC correlations, HSQC correlations, ^1^H-NMR splitting patterns and δ ^1^H- and ^13^C-values established the presence of two propyl groups between C-9 (9′) and C-11 (11′). The olefin proton H-8 showed HMBC correlations to the methylene C-10 and olefinic carbon C-3,3a. And the olefin proton H-8′ showed HMBC correlations to the methylene C-10′, olefinic carbon C-3′ and quaternary carbon 3a′. The olefin proton H-6′, H-7′ respectively exhibited HMBC cross peaks with C-7a′ and C-3a′ (Figure 2). The methine proton H-7 showed key HMBC correlations with C-4′ and C-3a′, and the methine proton H-6 showed key HMBC correlations with the carbonyl carbon C-1′ and quaternary carbon C-7a′, indicating that the two monomer units were linked by 6. 7a′- and 7. 3a′-bonds. Consequently, compound 6 was characterized as tokinolide A (**6**). Only a few special ^1^H-NMR signals of this compound had been previously reported [20]. The ^1^H- and ^13^C-NMR spectroscopic data for the compound are thus completely reported here for the first time.

**Figure 3:**
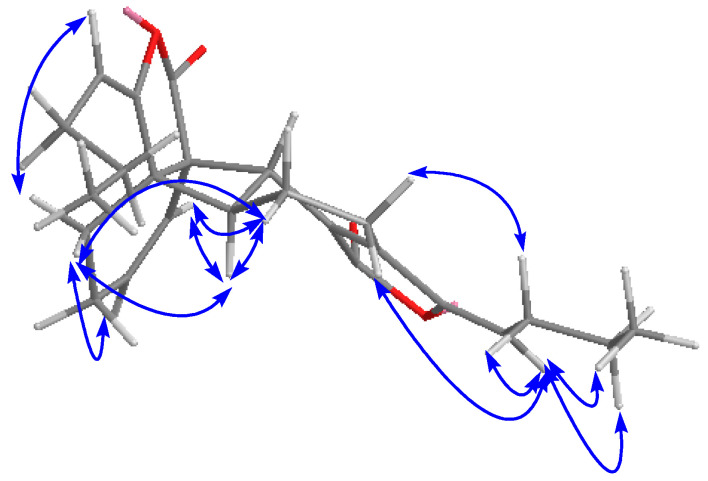
Key NOE correlations for compound **7**.

HRESIMS of compound **7** gave an [M + H]^+^ peak at *m*/*z* 381.2057, an [M + Na]^+^ one at *m*/*z* 403.1881 and an [2M + Na]^+^ one at *m*/*z* 783.3898, suggesting a molecular formula of C_24_H_28_O_4_. The IR spectrum suggested the existence of lactone (1760 cm^−1^) and cyclohexene groups (1700 cm^−1^). The UV spectrum showed an absorption maximum at 280 nm. Comparison of the ^1^H- and ^13^C-NMR data of **7** (Table 2) with those of **6** indicated that they had the similar overall structures. The methylene proton H-4′ showed key HMBC correlations with C-6 (Figure 2). NOE correlations between H-4′ and H-5/H-6 were observed (Figure 3). The above results suggested that the two monomer units were linked by 6.3a′- and 7.7a′-bonds. The relative configuration of **7** was assigned by the NOESY experiment combined with MM2 energy-minimized three-dimensional molecular modeling. The NOE correlations of H-4 with H-8, and H-8′ with H-4′/H-5′ established that they are on the same face of the molecule. Additional NOEs were observed between H-4′ and H-5/H-6, and also between H-7′ and H-5/H-6, indicating that the protons at C-4′, C-7′, C-5 and C-6 were on the same side of the ring system. Furthermore, the NOE correlations of H-8/H-4 and H-8′/H-4′ suggested a 3*Z*, 3′*Z*-configuration for the double bonds. Thus, compound **7** was elucidated with a structure as shown in Figure 1 and assigned as tokinolide C (**7**).

### 2.2. Cytotoxicity Assay

#### 2.2.1. Cytotoxic Activity of Compounds

Seven monomeric and dimeric phthalides isolated from the supercritical fluid CO_2_ extract of *Angelicae Sinensis* Radix, were evaluated against three cancer cell lines by MTT assays, and the results are summarized in Table 3. All isolated compounds were evaluated for their cytotoxic activities against the HepG2, HCT-8, and A549 cancer cell lines. After the treatment with compounds for 24 h, riligustilide (**4**) exhibited activity, with IC_50_ values ranging from 6.79 to 13.82 μM, tokinolide A (**6**) showed weak activity with IC_50_ values ranging from 27.79 to 34.34 μM, and tokinolide C (**7**) showed weak activity with IC_50_ values ranging from 30.92 to 55.84 μM against three cancer cell lines. No cytotoxic activity was observed for **1**–**3** and **5** (IC_50_ > 80 μM).

#### 2.2.2. Apoptosis-Inducing Activity 

Riligustilide (RLG, **4**), which exhibited potent cytotoxic activities against cancer cells, was next evaluated for its apoptosis-inducing activity. In three cancer cell lines, HCT-8 cells were the most sensitive to treatment with RLG, so they were selected to evaluate the possible mechanism of cytotoxic action of RLG. HCT-8 cells were incubated with the 10 μM test compound for 24 and 48 h, and then the cells were analyzed by means of flow cytometry with annexin V-propidium iodide (PI) double staining. 

**Figure 4:**
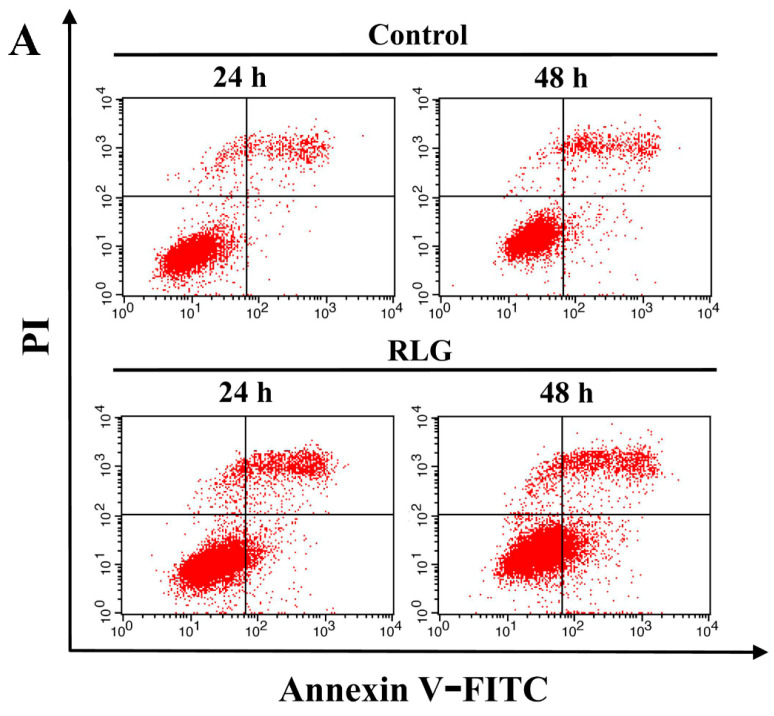

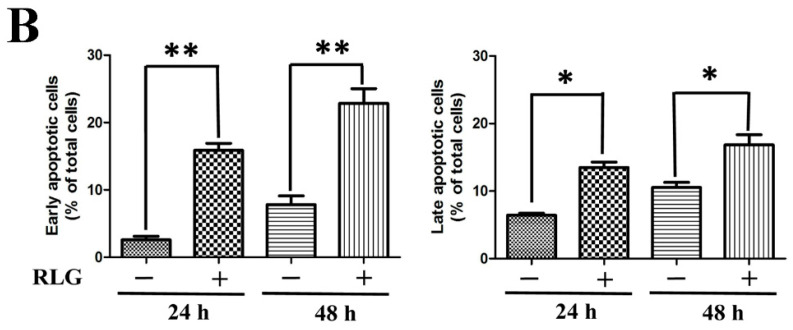
Riligustilide induced apoptosis against HCT-8 cells. (**A**) Representative apoptotic profile of HCT-8 cells treated with 10 μM RLG for 24 and 48 h by flow cytometry assay; (**B**) Statistical analysis of cell apoptotic rate after the treatment of RLG. The data are expressed as the means ± S.D. of three independent experiments with similar results. Student’s *t*-test was used for two group comparison. * *p* < 0.05; ** *p* < 0.01 *vs.* the control. “+” represents that 10 μM RLG was added, “−” represents control.

As shown in Figure 4, the ratio of early apoptotic cells (lower right) was increased after treatment with RLG in HCT-8 cells for 24 h (15.9% *vs.* 2.6% of control, *p* < 0.01) and 48 h (22.9% *vs.* 7.8% of control, *p* < 0.01), and that of late apoptotic cells (upper right) was also increased after 24 h (13.5% *vs.* 6.4% of control, *p* = 0.01) and 48 h (16.8% *vs.* 8.5% of control, *p* = 0.02). These results demonstrated that the cytotoxic activities of compound RLG against HCT-8 cells is due to inducing apoptotic cell death.

#### 2.2.3. Induction of Cell Cycle Arrest in G1 and S Phase

To investigate the effects of RLG on the cell cycle, HCT-8 cells were treated with the 5 μM test compound for 24 h and 48 h, respectively. After an incubation period, the cells were fixed and labeled with propidium iodide. The different phases of the cell cycle were analyzed by flow cytometry. 

**Figure 5:**
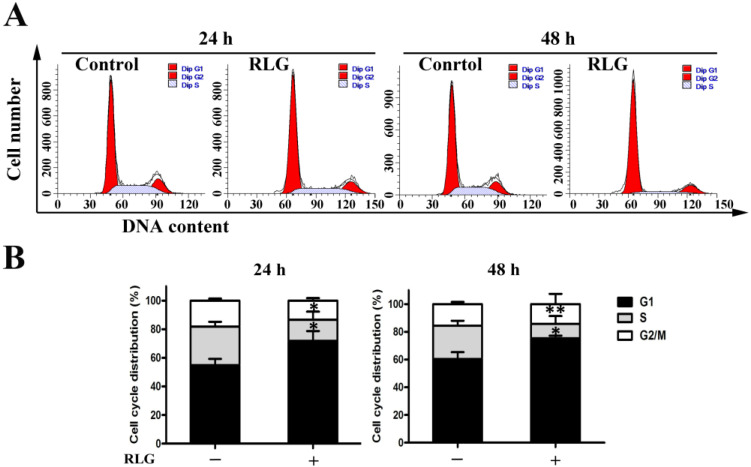
Effect of riligustilide on the cell cycle in HCT-8 cells. Cells were treated with riligustilide (5 μM) for 24 and 48 h. Then the cells were fixed and stained with PI to analyze DNA content by flow cytometry. (**A**) Representative histograms of one cell cycle analysis; (**B**) DNA content of the gated cells ± S.D. of three independent experiments. Student’s *t*-test was used for two group comparison. * *p* < 0.05; ** *p* < 0.01 *vs*. the control. “+” represents that 10 μM RLG was added, “−” represents control.

As can be observed from Figure 5, 24 h and 48 h treatment with RLG produced a significant increase of the proportion of cells in G1 phase (24 h: 71.78% *vs.* 54.86% of control, *p* = 0.03; 48 h: 75.42% *vs.* 60.41% of control, *p* = 0.02), along with the reduction of cells in S phase (24 h: 14.89% *vs.* 26.99% of control, *p* = 0.04; 48 h: 6.63% *vs.* 24.03% of control, *p* < 0.01), whereas the G2/M remain practically unchanged.

### 2.3. Neuroprotective Effect of Compounds

Compounds **1**–**7** were evaluated *in vitro* for their neuroprotective effects on neuron-like SH-SY5Y cells induced by glutamate, using the MTT method. All of the compounds tested were evaluated and given a corresponding inhibitory (%) (Table 4). Among the compounds tested, **1**, **3** and **6** at 10 μM exhibited significant effects against SH-SY5Y cells injured by 20 mM glutamate for 48 h (**1**: inhibition 27.1; **3**: inhibition 17.0; **6**: inhibition 22.2). The effect of compounds **1**–**7** on SH-SY5Y cells at 10 μM was also evaluated. As shown in Table 5, obvious cell damage effects on cell survival rate was observed when the cells were treated with compound **4** for 48 h, while no significant changes were observed on the viability of cells treated with other compounds. The cytotoxic effect of compound **4** on SH-SY5Y cells at 10 μM hampered the assessment of neuroprotection.

## 3. Experimental Section

### 3.1. General Procedures

NMR spectra were recorded on an Avance DRX-600 spectrometer (Bruker, Rheinstetten, Germany) operating at 600 (^1^H) and 150 (^13^C) MHz with TMS as an internal standard. HRESIMS were ontained on a 1260-6520 QT MS (Agilent, Santa Clara, CA, USA). UV data were recorded on a UV-2450 spectrophotometer (Shimadzu, Kyoto, Japan). IR spectra were measured on a FTIR-8400S spectrometer (Shimadzu, Kyoto, Japan). Column chromatography (CC) supports including silica gel (200–300 mesh, Haiyang Chemical Group Co. Ltd., Qingdao, China), and Sephadex LH-20 gel (25–100 μm; Pharmacia Fine Chemicals, Uppsala, Sweden) were also employed. A microplate reader (BMG FLUOStar OPTIMA, Ortenberg, Germany) was used to monitor the growth of the cells. TLC was carried out with glass precoated silica gel GF254 plates (Qingdao Marine Chemical Co. Ltd., Qingdao, China).

### 3.2. Plant Material

*Angelica Sinensis* Radix was purchased from Shanxi Huayang Pharmaceutical Company (Taiyuan, China) and authenticated by Xue-Mei Qin, Shanxi University, before preparation. A voucher specimen was deposited in the Modern Research Center for Traditional Chinese Medicine of Shanxi University, China.

### 3.3. Extraction and Isolation

A supercritical fluid extraction system (HA121-50-01, Huaan Scientific Co. Ltd., Jiangsu, China) was used for the extraction. The parameters, including pressure, temperature, static time, had been optimized based on the yields of extract. *Angelica Sinensis* Radix (8 kg) was extracted under the following conditions: pressure 20 MP; temperature 50 °C; static time 3 h; solvent flow rate 15 kg/h, and 81.2 g extract was thus obtained. The extract was separated into 11 fractions (A–K) by silica gel CC, eluting with a step gradient of petroleum ether-ethyl acetate from 100:0 to 0:100 (*v*/*v*). Fraction B (3.9 g) was was chromatographed on Sephadex LH-20 gel CC by elution with CHCl_3_–MeOH (2:1) to afford compound **3** (98 mg). Fraction C (4.8 g) was subjected to SiO_2_ CC to give six fractions (C_1_–C_6_). Fraction C_2_ (1.5 g) was chromatographed on Sephadex LH-20 gel CC to afford compounds **1** (30.0 mg) and **2** (23.0 mg). Fraction D (2.9 g) was separated using Sephadex LH-20 gel CC by elution with CHCl_3_–MeOH (2:1) to afford six subfractions (D_1_–D_6_). Fraction D_4_ (0.8 g) was subjected to HPLC using 80% aqueous MeOH (4 mL/min) to produce compound **5** (45.5 mg, t_R_ = 9.0 min). Fraction F (5.7 g) was further chromatographed on silica gel with an petroleum ether-ethyl acetate solvent system to provide seven subfractions (F_1_–F_7_). Fraction F_4_ (1.6 g) was purified using Sephadex LH-20 gel CC by elution with CHCl_3_–MeOH (2:1) to afford four subfractions (F_4A_–F_4D_). Fraction F_4C_ (0.7 g) was further purified using HPLC (MeOH–H_2_O = 70:30, 4 mL/min) to yield **6** (30.7 mg, t_R_ = 45.59 min) and **7** (23.8 mg, t_R_ = 61.15 min). Fraction H (2.5 g) was further chromatographed on silica gel with an petroleum ether-ethyl acetate solvent system to provide ten subfractions (H_1_–H_10_). The purification of subfraction H_8_ (0.5 g) by Sephadex LH-20 gel CC with CHCl_3_–MeOH (2:1) afforded compound **4** (89.2 mg).

### 3.4. Product Characterization

*Tokinolide C* (**7**): pale yellow oil; UV (MeOH) λ_max_ (log ε) 280 nm ; IR υ_max_ 1760, 1700 cm^−1^; For ^1^H-NMR (600 MHz, CDCl_3_) and ^13^C-NMR (150 MHz, CDCl_3_) spectroscopic data, see Table 2; HRESIMS *m*/*z* 381.2057 [M + H]^+^ (calcd for C_24_H_28_O_4_, 381.1980).

### 3.5. Cytotoxicity Assay

#### 3.5.1. Cell Line Cultures

The A549 (human lung carcinoma), HCT-8 (human colon carcinoma), Hep G2 (human liver carcinoma) cell lines were obtained from Institute of Materia Medica, Chinese Academy of Medical Sciences and Peking Union Medical College (Beijing, China). Cells were cultured in RPMI 1640 culture medium supplemented with 10% fetal bovine serum, 100 units/mL penicillin, and 100 μg/mL streptomycin at 37 °C, 5% CO_2_.

#### 3.5.2. Cell Viability Assay

Cell viability was determined by the 3-(4,5-dimethylthiazol-2-yl)-2,5-diphenyltetrazolium bromide (MTT) assay. Cells were plated on a 96-well plate at 3 × 10^3^ cells/well and exposed to the test compounds (1, 5, 10, 20, 40, 80 μM) for 24 h. Cultures were also treated with 0.1% DMSO as the vehicle control. After 24 h of treatment, 10 μL of MTT solution (5 mg/mL) was added to each well and the plates were incubated for 4 h at 37 °C. The supernatant was then removed from formazan crystals, and 100 μL of DMSO was added to each well. The absorbance at 570 nm was read using a M200 PRO microplate reader (Tecan). The cell viability was calculated by dividing the mean optical density (OD) of compound containing wells by that of 0.1% DMSO-control wells [21]. Doxorubicin and DMSO were used as positive and negative controls, respectively.

#### 3.5.3. Cell Apoptosis Assay

The apoptotic cells were measured by flow cytometry using the Annexin V-FITC and PI double staining kit (Nanjing Keygen Biotechnology Co. Ltd., Nanjing, China). Briefly, HCT-8 cells were plated with 1 × 10^5^ cells/well at a 6-well plate and cultured overnight for attachment. Cells were treated with test compound at 10 μM for 24 h and 48 h respectively. After an incubation period, the cells were collected, washed with PBS, and resuspended in 200 μL of binding buffer containing 5 μL of Annexin V-FITC and 5 μL of PI for 20 min in the dark. The samples were immediately analyzed by a FACSCalibur flow cytometry (BD Biosciences, San Jose, CA, USA). About 10,000 events were recorded for each sample.

#### 3.5.4. Cell Cycle Assay 

The DNA content of cells in the G0/G1, S, and G2/M phases can be determined by flow cytometry. Briefly, HCT-8 cells were plated with 1 × 10^5^ cells/well at a 6-well plate and cultured overnight for attachment. Cells were treated with test compound at 5 μM for 24 h and 48 h respectively. After an incubation period, the cells were collected, centrifuged and fixed with ice-cold ethanol (70%) overnight at 4 °C. Cells were centrifuged at 1000 rpm for 5 min to remove all the ethanol. Each cell pellet were then treated with lysis buffer containing RNAse A and 0.1% Triton X-100, and then incubated with PI for 30 min at room temperature in the dark. Then, cells were washed in PBS twice. Samples were analyzed on a FACSCalibur flow cytometer (BD Biosciences). About 20,000 events were recorded for each sample.

### 3.6. Neuroprotective Effects Assay

Neuroprotective effects were assayed in SH-SY5Y cells injured by glutamate as reported previously [22]. SH-SY5Y cells were obtained from Institute of Materia Medica, Chinese Academy of Medical Sciences and Peking Union Medical College. SH-SY5Y cells at a density of 1 × 10^4^ cells per well in 96-well plates were cultured in DMEM media supplemented with 10% fetal bovine serum. After 24 h incubation, the medium of model group was changed to basic medium with 20 mM glutamate for 48 h. Tested compounds (10 μM) dissolved in dimethyl sulfoxide (DMSO) were added to each well for >1000 fold dilution in the model medium at the same time. *N*-Methyl-d-aspartate (NMDA) receptor antagonist dizocilpine (MK-801) was used as positive control. Each sample was tested in triplicate.

Besides, the cytotoxic effect or proliferation effect of these tested compounds on SH-SY5Y cells was also evaluated in the present research. Briefly, cells were plated on a 96-well plate at 1 × 10^4^ cells per well. After 24 h incubation, the cells were exposed to the test compounds (10 μM). Cultures were also treated with 0.1% DMSO as the vehicle control. Each sample was tested in triplicate.

After the incubation at 37 °C in 5% CO_2_ for 48 h, 10 μL of MTT (5 mg/mL) was added to each well and incubated for another 4 h, then liquid in the wells was removed. DMSO (100 μL) was added to each well. The absorbance was recorded on a microplate reader at a wavelength of 570 nm. The survival rate of SH-SY5Y cells was evaluated, and the inhibition (%) (Table 4) were obtained using the following formula:

Inhibition (%) = [(OD_(sample)_ − OD_(model)_)/OD_(control)_ − OD_(model)_] × 100

### 3.7. Statistical Analysis

Data were presented as means ± S.D. Statistical analysis was performed using the student’s *t*-test method or one-ANOVA followed by Dunnett’s test when appropriate. Values of *p* < 0.05 were considered statistically significant.

## 4. Conclusions

Seven monomeric and dimeric phthalides, including a new compound, were isolated from the *Angelicae Sinensis* Radix. Evaluation of their cytotoxic activity against three human cancer cell lines established that riligustilide (**4**) exhibited potent cytotoxic activity against cancer cells, and this was demonstrated mainly by the induction of apoptosis and cell cycle arrest detected by flow cytometry. In addition, *in vitro* neuroprotective assays showed that compounds **1**, **3** and **6** exhibited significant effects against SH-SY5Y cells injured by glutamate at a concentration of 10 μM, suggesting that these compounds could serve as candidates for potential application in the treatment of neurodegenerative diseases.

## Figures and Tables

**Table 1:** ^1^H, ^13^C and HMBC correlation data for tokinolide A (**6**) ^a^.

Position	δc, Type	δ_H_, (*J* in Hz)	HMBC	Position	δc, Type	δ_H_, (*J* in Hz)	HMBC
1	169.5, qC			1′	175.4, qC		
3	149.0, qC			3′	151.4, qC		
3a	151.4, qC			3a′	48.2, qC		
4	17.8, CH_2_	2.56 m, 2.40 m	5, 3a, 7a	4′	29.7, CH_2_	1.94 m, 2.03 m	5′, 7, 3a′, 7a′
5	20.1, CH_2_	1.70 m, 2.12 m	4, 6, 7, 7a′	5′	21.0, CH_2_	2.10 m	7′, 3a′, 4′, 6′
6	40.2, CH	2.90 m	4, 5, 7, 1′,7a′	6′	131.1, CH	6.11 m	4′, 5′, 7a′
7	34.1, CH	3.23 d (9.4)	4′, 6, 3a, 7a, 3a′	7′	124.6, CH	6.00 d (9.8)	5′, 3a′, 7a′
7a	125.3, qC			7a′	46.4, qC		
8	112.1, CH	5.21 t (7.9)	3, 3a, 10	8′	106.9, CH	4.66 dd (7.1, 8.3)	3′,3a′, 10′
9	27.9, CH_2_	2.36 m	3, 8, 10, 11	9′	27.2, CH_2_	2.08 m, 1.90 m	3′, 8′, 10′, 11′
10	22.4, CH_2_	1.49 m	8, 9, 11	10′	22.5, CH_2_	1.25 m	8′, 9′,11′
11	13.7, CH_3_	0.95 t (7.4)	9, 10	11′	13.5, CH_3_	0.80, (7.4)	9′,10′

^a^ Data obtained on a Varian 600 MHz instrument in CDCl_3_.

**Table 2:** ^1^H, ^13^C, HMBC and NOESY correlation data for tokinolide C (**7**) ^a^.

Position	δc, Type	δ_H_, (*J* in Hz)	HMBC (H → C)	NOESY
1	168.7, qC			
3	148.9, qC			
3a	153.5, qC			
4	19.0, CH_2_	2.21 m, 2.57 m	3, 5, 6, 3a, 7a	8, 9
5	21.9, CH_2_	1.98 m	6, 3a	6, 4′
6	37.1, CH	2.99 dt (10.4, 8.5)	5, 7, 3a′	5, 4′, 7′
7	37.0, CH	3.39 d (8.5)	6, 3a, 7a, 7a′	
7a	124.5, qC			
8	112.2, CH	5.24 t (7.9)	3, 3a, 10	4, 9, 10, 11
9	27.2, CH_2_	2.37 m	3, 8, 10, 11	4, 8, 10, 11
10	22.34, CH_2_	1.51 m	8, 9, 11	8, 9, 11
11	13.56, CH_3_	0.96 t (7.4)	9, 10	8, 9, 10
1′	176.9, qC			
3′	153.4, qC			
3a′	48.5, qC			
4′	24.1, CH_2_	1.77 m, 1.88 m	6, 3′, 6′, 7a′	6′, 7′, 8′, 6, 5
5′	20.6, CH_2_	1.86 m	3a′, 4′, 6′	8′
6′	133.8, CH	6.11 ddd (9.9)	4′, 5′, 7a′	4′, 7′
7′	121.0, CH	5.77 dd (1.7, 9.9)	4′, 3a′	4′, 6, 5
7a′	48.3, qC			
8′	103.9, CH	4.88 t (7.9)	3′, 3a′, 10′	4′, 9′, 10′, 11′
9′	28.0, CH_2_	2.18 m	3′, 8′, 10′,11′	8′, 10′, 11′
10′	22.6, CH_2_	1.44 m	8′, 9′, 11′	8′, 9′, 11′
11′	13.7, CH_3_	0.92 t (7.4)	9′, 10′	8′, 9′, 10′

^a^ Data obtained on a Varian 600 MHz instrument in CDCl_3_.

**Table 3:** Cytotoxic activities of the compounds against three human cancer lines.

Compound	IC_50_ (μM) ^a^		
	A549	HCT-8	HepG2
**1**	>80	>80	>80
**2**	>80	>80	>80
**3**	>80	>80	>80
**4**	13.82 ± 2.23	6.79 ± 1.14	7.92 ± 1.38
**5**	>80	>80	>80
**6**	47.63 ± 4.51	55.84 ± 5.99	30.92 ± 2.36
**7**	34.34 ± 3.80	27.79 ± 3.42	32.54 ± 2.69
**Doxorubicin ^b^**	0.28 ± 0.05	1.55 ± 0.45	0.65 ± 0.11

Values are the mean ± S.D. for three separate experiments; ^a^ Compound concentration required to inhibit cell growth by 50%. Cells were treated with test samples (1–80 μM) for 24 h; ^b^ Doxorubicin was used as positive control, and DMSO was negative control.

**Table 4:** Survival rate of SH-SY5Y cells injured by glutamate ^a^.

Group	Cell Survival Rate (% of Control)	Inhibition (% of Model)
Control	100 ± 9.4	
Model	60.2 ± 2.3 ^##^	
MK-801 ^b^	72.8 ± 5.9 **	31.7
**1**	71.0 ± 4.1 **	27.2
**2**	63.6 ± 4.9	8.3
**3**	67.0 ± 2.1 *	17.0
**4**	50.7 ± 7.0	−24.0
**5**	59.3 ± 4.2	−2.4
**6**	69.0 ± 7.6 *	22.2
**7**	62.5 ± 2.0	5.6

^a^ All the compounds were tested at 10 μM; ^##^ *p* < 0.01 *vs.* control; * *p* < 0.05; ** *p* < 0.01 *vs.* model group. One-way analysis of variance was used, *n* = 3; ^b^ MK-801 was used as positive control.

**Table 5:** The effects of compounds **1**–**7** on SH-SY5Y survival rate ^a^.

Group	Cell Survival Rate (% of Control)	Group	Cell Survival Rate (% of Control)
Control	100.0 ± 10.5	**4**	57.4 ± 4.9 **
**1**	99.6 ± 5.9	**5**	97.9 ± 5.2
**2**	96.6 ± 2.3	**6**	90.9 ± 14.8
**3**	100.8 ± 13.8	**7**	93.0 ± 5.4

^a^ All the compounds were tested at 10 μM for 48 h; ** *p* < 0.01 *vs.* control; One-way analysis of variance was used, *n* = 3.
