# Correction: Gong et al. Neuroprotective and Cytotoxic Phthalides from *Angelicae Sinensis* Radix. *Molecules* 2016, *21*, 549

**DOI:** 10.3390/molecules28237814

**Published:** 2023-11-28

**Authors:** Wenxia Gong, Yuzhi Zhou, Xiao Li, Xiaoxia Gao, Junsheng Tian, Xuemei Qin, Guanhua Du

**Affiliations:** 1 Modern Research Center for Traditional Chinese Medicine, Shanxi University, No.92, Wucheng Road, Taiyuan 030006, China; 2 Institute of Materia Medica, Chinese Academy of Medical Sciences & Peking Union Medical College, Beijing 100050, China

**Error in Figure**

In the original publication [1], there was a mistake in **Figure 5** as published. **The images shown for the control group in 24 h are incorrect; the images for the control group in 48 h were inadvertently inserted because the two images are similar**. The corrected **Figure 5** appears below. The authors apologize for any inconvenience caused and state that the scientific conclusions are unaffected. This correction was approved by the Academic Editor. The original publication has also been updated.

## Figures and Tables

**Figure 5:**
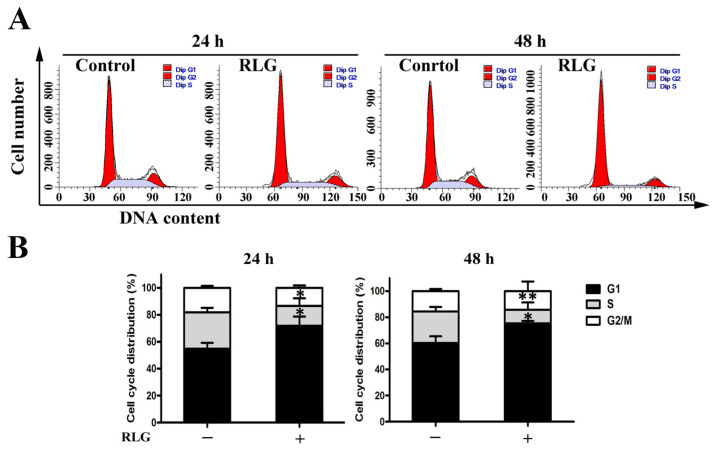
Effect of riligustilide on the cell cycle in HCT-8 cells. Cells were treated with riligustilide (5 μM) for 24 and 48 h. Then the cells were fixed and stained with PI to analyze DNA content by flow cytometry. (**A**) Representative histograms of one cell cycle analysis; (**B**) DNA content of the gated cells ± S.D. of three independent experiments. Student’s *t*-test was used for two group comparison. * *p* < 0.05; ** *p* < 0.01 vs. the control. “+” represents that 10 μM RLG was added, “−” represents control.
